# Engineering of bio-mimetic substratum topographies for enhanced early colonization of filamentous algae

**DOI:** 10.1371/journal.pone.0219150

**Published:** 2019-07-05

**Authors:** Ali Khoshkhoo, Andres L. Carrano, David M. Blersch, Kamran Kardel

**Affiliations:** 1 Department of System Science and Industrial Engineering, Binghamton University, Binghamton, New York, United States of America; 2 Department of Manufacturing Engineering, Georgia Southern University, Statesboro, Georgia, United States of America; 3 Department of Biosystems Engineering, Auburn University, Auburn, Alabama, United States of America; VIT University, INDIA

## Abstract

This work reveals a set of surface topography parameters that are significant for algal attachment to natural rock substrata. Topography analysis of rock surfaces from a stream identifies three descriptive areal parameters (*S*_*mr*_, *S*_*v*_, and *S*_*a*_) that correlate with the presence of natural periphyton community. A method was developed and validated to reverse engineer and manufacture artificial substrata with topographic complexity defined by these parameters, using computational modeling and additive manufacturing. Results from colonization experiments with filamentous algae show statistically significant increases in early biomass accrual rates on substrata with higher values of *S*_*a*_ and *S*_*v*_ parameters and lower values of *S*_*mr*_ parameter. These results suggest that manipulation of the level of roughness (peak-to-valley distance and material ratio above the mean) and the distribution of hill and dale sequences can control initial colonization locations and biomass accrual rates, presumably by enhancing growth and recruitment of cells from the overlying flow into protected refugia spaces. As such, these findings provide an approach for optimizing the design of substratum for increased early biomass productivity for attached growth algae cultivation systems.

## Introduction

The cultivation of filamentous algae on substrata presents many advantages over other approaches to algae cultivation, including fast rates of growth, rapid uptake kinetics at low concentrations of dissolved growth factors, and ease of harvest and recovery of biomass at a high solids content [1]. The cultivation of attached filamentous algae in technologies such as the algal turf scrubber (ATS) employs these advantages for recovery of pollutant nutrients from impacted waters, and many applications of ATS technology have been investigated for pollutant management from stormwater and agricultural wastewaters [2–6]. In ATS systems, the algal community is typically dominated by large filamentous species that attach to a substratum surface, so that the active algae remain in place as nutrients come to them via flow. The filamentous form conveys advantages of a large active surface area for uptake of dissolved constituents and interception of light, thereby maximizing growth by modulating potential limiting factors. In addition, because the biomass remains attached to a surface, it is easily recovered by low-cost methods of harvesting and dewatering. Due to their high growth rates, unique constituent biomolecules, and rapid regeneration capabilities, cultivated filamentous algae constitute a promising source of biomass for bio-economic materials and a promising process approach for water remediation [1].

A key basis for the design of ATS systems is the artificial solid substratum to which filamentous algal species attach, critical for the regeneration of the algal turf following repeated harvesting [7]. The early colonization of a bare substratum at startup is arguably a critical step in algae community formation that contributes to species presence within an ATS turf. Despite reporting on applications of ATS technology over many years [7], there is limited published work regarding the design of substratum in ATS systems, except for some reporting on the effects of 3D substratum designs on productivity [3]. All the algal species that might colonize an ATS substratum have a natural context for their activity, where different species demonstrate preferences for different materials (e.g., rock, wood, sand) [8] or different levels of roughness [9] in flowing water systems. Therefore, much can be learned from characterizing the topography of natural surfaces successfully colonized by algae in flowing waters.

Microtopography is known to have a strong influence on colonization and growth kinetics of cells in an attached algal community. Factors such as cell-topographic feature size matching [10], boundary layer hydrodynamics [11–13], and material surface chemistry [14] have been demonstrated to affect individual cell kinetics of colonization and growth. Some combination of these factors has been shown to affect the structure of the periphyton community [8–10, 14–17], through competitive exclusion and succession principles of community organization. Thus, the design of microtopography for substratum surfaces affords an approach for selection of algal taxa from a colonizing community through modulation of colonization and growth dynamics.

New methods for manufacturing microtopography on surfaces have recently become available, including laser surface texturing [18], abrasive jet machining [19], and etching [20]. Most recently, Additive Manufacturing (AM), or 3D printing, provides precise fabrication of surface topography at a low cost through material deposition. Because 3D printing uses a slicing algorithm to layer a solid model, it offers the ability to fabricate unique geometries that are impossible through other typical fabrication methods [21–22]. Despite its prevalence, however, AM has rarely been used to design surfaces for algal cultivation, although feasibility has been demonstrated in field and laboratory trials for early colonization [21, 23]; growth rates [17, 24–25], and community structure [9]. Most studies used substrate topographies, however, composed of repeated idealized shape elements, such as hemispheres, and none addressed randomized surface topographies that mimic natural substrata. Quantifying the topographic characteristics of natural colonized surfaces gives the opportunity to uncover the physical features that control colonization patterns of algae on substrata, which may then be useful for design of productive material surfaces.

The overall goal of this study is to understand the effect of engineered substratum topography that is mimetic of natural surfaces on the colonization and growth rates of attached filamentous algae in a flow environment, with the motivation to design high-productivity substrata for biomass cultivation. The specific objectives of this study were to model surface topographies of natural rock surfaces that correlate with living periphyton in streams and to use those models to test for algal colonization rates on manufactured substrata with similar topographic parameter descriptors.

## Materials and methods

**Fig 1:**
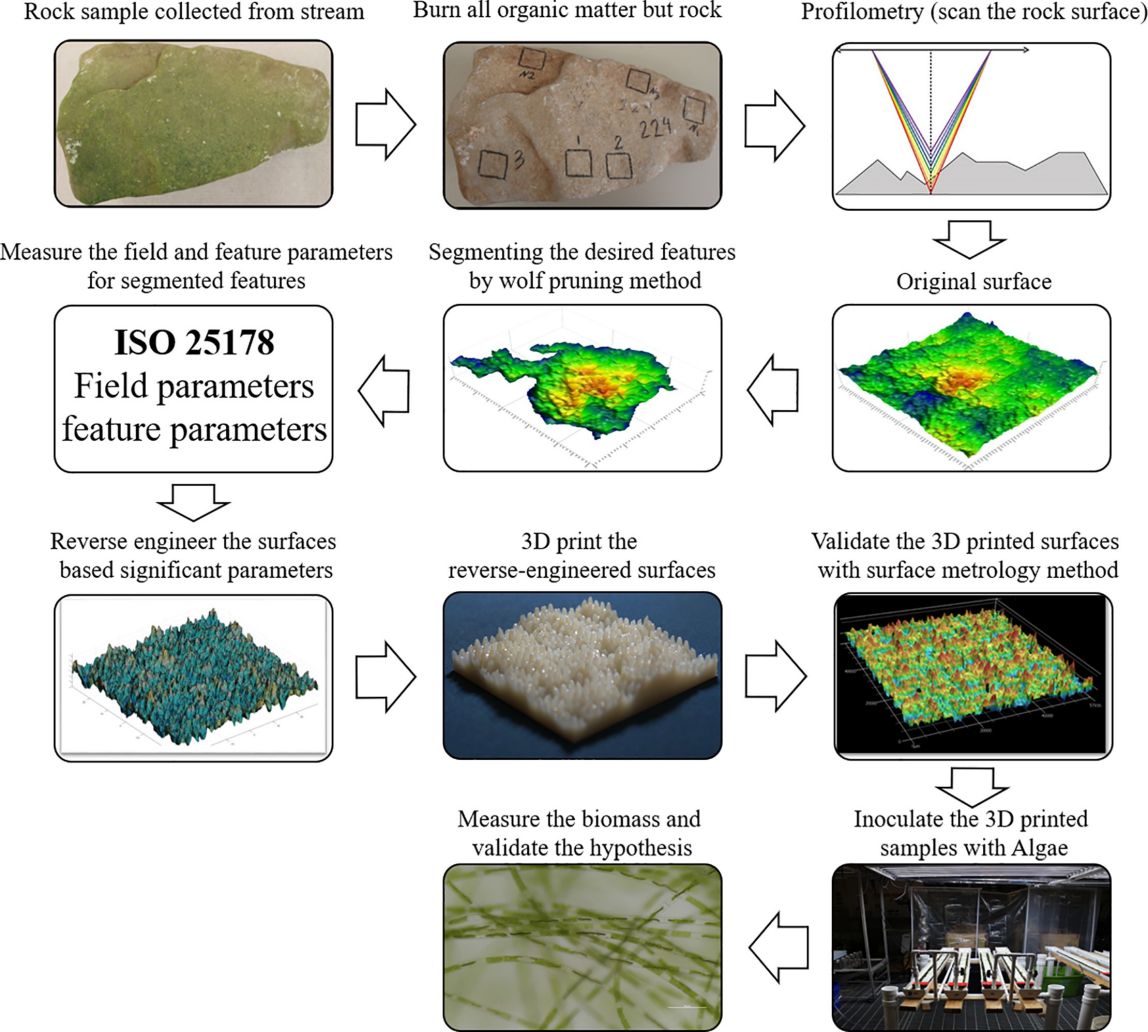
Schematic of the methodology workflow for reverse engineering and testing of natural substrata.

As an overview, the approach for this work was to analyze the surface characteristics of periphyton-colonized rocks from a natural stream for descriptive surface parameters that correlate with algal presence. Substrata with pseudo-randomized surfaces were modeled to match these parameters at different levels and manufactured via AM for the study of the early colonization of attached filamentous algae in a flow environment. An overview of the methodology pursued in this work is depicted in the general workflow in Fig 1. The broad sequence of steps included: (a) retrieval of natural rock specimens from streams, and demarcation of areas where periphyton growth was either present or absent; (b) organic matter removal via combustion in a muffle furnace; (c) scanning and profilometry of demarcated areas; (d) analyses of areal feature parameters, and correlation with the presence or absence of periphyton; (e) segmentation and extraction of significant features (i.e., dales and hills); (f) statistical analysis of field and feature parameters and fitness to a probability distribution function; (g) pseudo-randomized generation of surface computer models with parametric matching; (h) fabrication of experimental substratum surface tiles with material jetting processes; (i) profilometer-based validation of the fabricated model fidelity; (j) exposure of fabricated tile replicates to a colonizing algal community in a floway photobioreactor; and (k) measurement of biomass accrual rates on tile replicates.

### Natural surface analysis (steps *a-e*)

Rock specimens were collected from Chewacla Creek in Chewacla State Park, Auburn, Alabama (32°32'51.0"N 85°28'53.7"W) on July 7^th,^ 2015. The collection process was authorized by the Department of Conservation and Natural Resources in the State of Alabama. The specimens were collected by considering two criteria: (*i*) their flow-facing surfaces being flat enough (*S*_*z*_ range: [426.45 μm-779.17 μm]) to be scanned with a high-resolution white-light profilometer (Z-range 800 μm), and (*ii*) their flow-facing surfaces containing both colonized and bare areas. Specimens with a high visual contrast between colonized and bare surfaces were targeted. The specimens were collected from the same cross-section of the creek, at which there was a USGS stream gage (USGS 02418760) that records information on water discharge and water level every fifteen minutes [26]. At the time of sampling, the mean gage height was 0.56 ± 0.04 m, while water discharge was 0.52 ± 0.33 m^3^/s. The mean water temperature during the collecting the samples was 27.90±1.30 ^o^C with an average daytime light intensity of 224 μmol/m^2^/s at the surface of the water, as measured with a quantum flux meter (LI-250 Light Meter and LI-190 Quantum Sensor, LI-COR Biosciences, Lincoln, Nebraska, USA). The pH and conductivity of the water were measured with a handheld combination pH/EC probe (HI 98130, Hanna Instruments, Woonsocket, RI), and averaged 7.40 ±0.20 and 0.14 ±0.05 mS/cm, respectively. The local stream velocity was measured with a Model 2100 current flow velocity meter (Swoffer Instruments, Inc., Tukwila, WA) and averaged 0.25 ±0.10 m/s. Both dissolved P and N concentrations were moderately low (PO_4_-P: 0.08 ±0.02 mg l^-1^; NO_3_-N: 0.53 ±0.18 mg l^-1^, n = 5), as measured with a YSI 9500 field photometer (YSI Inc., Yellow Springs, Ohio).

Ten specimens of random mineral makeup and formation but with the desirable flow-facing surface topography were retrieved. On average, four squared areas (1 cm^2^) per specimen, forty in total, with various levels of algal attachment were identified and marked with graphite pencil immediately after collection. Standard dry ashing protocols [27] were used to remove material; the specimens were placed inside a furnace at 575°F for four hours to burn off all organic matter attached to the surface and allowed to cool for at least 24 hours. Following that, marked areas were measured for microscale topography with a structured light profilometer (ST400, Nanovea, Irvine, California) with a resolution of 20 nm and using a 5 μm step size in both X and Y directions and an acquisition rate of 100 Hz. Those areas where missing points accounted for less than 10% of the total raw data were kept. For the remaining ten specimens, the raw profilometry data was processed with Mountains (Digital Surf, Besancon, France) surface imaging and metrology software and used for parameter calculation and topography imaging.

To determine the need for feature segmentation (i.e., extraction of the desired features from the surface), a preliminary experiment with replicated pseudo-randomized 3D-printed textures was conducted in floway bioreactor for three weeks to single out the features (hills or dales) of interest for algal communities. It was observed that the algal attachment predominated in dales rather than hills and this is consistent with previous findings in the literature [28]. Therefore, dales were selected for segmentation as the features of interest.

This work followed the 5-step feature characterization established in ISO 25178–2 [29] consisting of the following steps: selection of the type of texture feature, segmentation, determination of significant features, selection of feature attributes, and quantification of feature attribute statistics (steps e and f, Fig 1). Wolf pruning [29] was used for pattern recognition and segmentation of significant and non-significant surface topographies. Since Wolf pruning produces different counts of features at various thresholds, the recommended 10% of *S_z_* (maximum height of the surface) was used as the threshold. This pruning of the data retained all data elements below the 10% watershed value.

**Fig 2:**
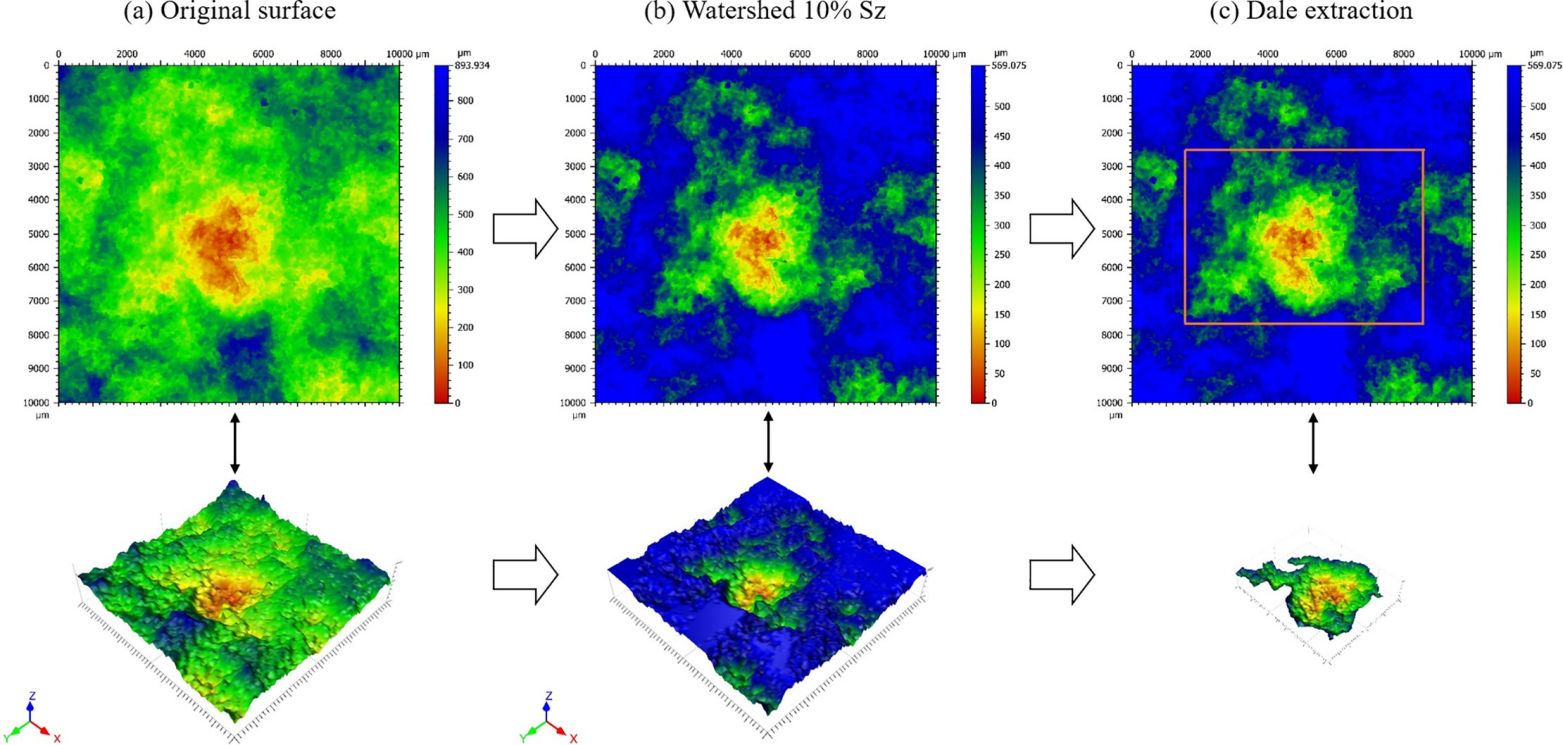
Feature segmentation and Wolf-pruning.

Once the colonized and bare areas were scanned, forty-two dales, twenty-one colonized and twenty-one bare, were selected. Each rock specimen had, on average, two colonized and two bare dales. The selected dales were extracted, and the parameters were calculated on those features. This feature recognition and segmentation as performed on one of the specimens is illustrated in Fig 2.

### Results of natural surface analysis and surface engineering (steps f-i)

Using profilometry after the 5-step feature characterization data, twenty-six areal surface parameters [29], as they presented in S1 Appendix, describing various aspects of surface topography were calculated and compared between colonized and bare dales using ANOVA tests. These included parameters from each of the surface texture classifications, such as feature parameters (i.e., *S*_*10z*_ , *S*_*5v*_), height parameters (i.e., *S*_*q*_, *S*_*sk*_,, *S*_*ku*_, *S*_*p*_, *S*_*v*_, *S*_*z*_, *S*_*a*_), functional parameters (i.e., *S*_*mr*_, *S*_*mc*_ , *S*_*xp*_), spatial parameters (i.e., *S*_*al*_, *S*_*tr*_, *S*_*td*_ ), functional volumetric parameters (i.e., *V*_*m*_, *V*_*v*_, *V*_*mp*_, *V*_*mc*_, *V*_*vc*_, *V*_*vv*_), and functional stratified parameters (i.e., *S*_*k*_, *S*_*pk*_, *S*_*vk*_, *S*_*mr1*_, *S*_*mr2*_). Results for the screening for significant parameters on rock surfaces is presented in Table 1. Among the chosen surface parameters, the areal material ratio (*S*_*mr*_), maximum pit height (*S*_*v*_), and arithmetical mean of the absolute of the ordinate values (*S*_*a*_) were found significant (α = 0.05) to explain the difference between the colonized and bare areas within the same specimen’s surface (Table 1). The *S*_*mr*_ (Eq 1) is the areal material ratio (expressed as a percentage) of the cross-sectional area of the surface at an average height relative to the evaluation cross-sectional area. The *S*_*v*_ is the lowest point found on the surface. The *S*_*a*_ (Eq 2) indicates significant deviations in the texture characteristics.

$$S_{mr}=\left(\frac{A(m)}{A(N)}\right)\times 100$$ (1)

$$S_{a}=\frac{1}{A}\iint_{A}^{}|z(x,y)|dxdy$$ (2)

where *A* is the area of the measured surface, *z(x*,*y)* is the height at the point *(x*, *y)*, *A (m)* is the area of the cross-sectional area of the surface at mean height *m*, and *A (N)* is the cross-sectional evaluation area.

**Table 1:** Significant surface texture parameters of colonized and bare areas.

Classification	Parameter	Description	Colonized		Bare		p-value
			Mean	Standard deviation	Mean	Standard deviation	
**Height parameters**	*S*_*v*_ *(μm)*	maximum pit height	203.94	49.14	163.63	55.86	0.01
	*S*_*a*_ *(μm)*	arithmetical mean of the absolute of the ordinate values	46.63	12.09	38.24	10.94	0.03
**Functional Parameters**	*S*_*mr*_ *(%)*	areal material ratio	0.001	0.001	0.004	0.002	<0.01

In addition, it was observed that the depth of the colonized dales (203.9 *μ*m) are significantly greater than that of the non-colonized dales (163.6 *μ*m), but there is no difference found between the height of the colonized (207.2 *μ*m) and non-colonized hills (192.6 *μ*m). The full statistical results are provided in the S1 Data.

In order to validate the results of the rock surface analysis, replicates of irregular surfaces with specified *S*_*mr*_, *S*_*v*_ and *S*_*a*_ values (Table 2) were modeled by the Pearson distribution [30–32] in Matlab and fabricated as growth tiles (50 x 50 x 7 mm) with an Objet30 3D printer (Stratasys Ltd., Eden Prairie, MN) using a 28 μm layer thickness. The material used to print the tiles is Objet Vero/white/plus Rgd835, which is a commercial resin provided by Stratasys and consists primarily of acrylic (<30%) and Isobornyl acrylate (< 25%).

**Table 2:** Experimental design parameters and levels.

	Average of targeted values		Average of actual values		Average error (%)	
Parameter	Level 1	Level 2	Level 1	Level 2	Level 1	Level 2
***S***_***mr***_ ***(%)***	30.0%	52.0%	31.5%	49.0%	5.2%	5.7%
***S***_***v***_ ***(mm)***	6.6 mm	8.4 mm	8.67 mm	11.2 mm	31.3%	33.3%
***S***_***a***_ ***(mm)***	0.7 mm	2.1 mm	0.91 mm	2.8 mm	30.0%	33.3%

The Pearson’s system of frequency curves are capable of generating a density function with given properties if its first four moments are known, i.e., the mean, standard deviation, skewness, and kurtosis [32]. The Pearson distribution was used to generate surfaces with precise, targeted first four moments values approaching the specific measured texture parameters from the natural surfaces. Since the density functions in this study are nondimensionalized (independent of dimensions and scales), they are dependent only on skewness and kurtosis of the surface.

Based on the data (i.e., first four moments) obtained from the rock surfaces scanned with the profilometer, the skewness and kurtosis values for both colonized and bare areas were approximately -0.3 and 3.0 (close to the values in a normally distributed surface), respectively. Thus, the skewness and kurtosis values for the reverse-engineered surfaces were kept as -0.3 and 3.0, respectively. With these constrained skewness and kurtosis values, the criterion *k*, which designates the density function type, was calculated as -0.256, which corresponds to the Pearson Type I. The *S_v_* value for the generated surface was targeted by multiplying a constant, *h*, with the pseudo-random numbers generated by the Pearson function for the z-coordinates of surface data to control the maximum height of the surface. The value of *h* was selected so that the maximum height of the samples would be less than or equal to flow depth in the bioreactor. The *S*_*a*_ values were obtained by changing the standard deviation of the pseudo-random numbers. Material ratio (*S*_*mr*_) was obtained by applying micro-patterned depression patterns to modulate the values of the material ratio and approach specific *S*_*mr*_ values for the generated surfaces.

**Fig 3:**
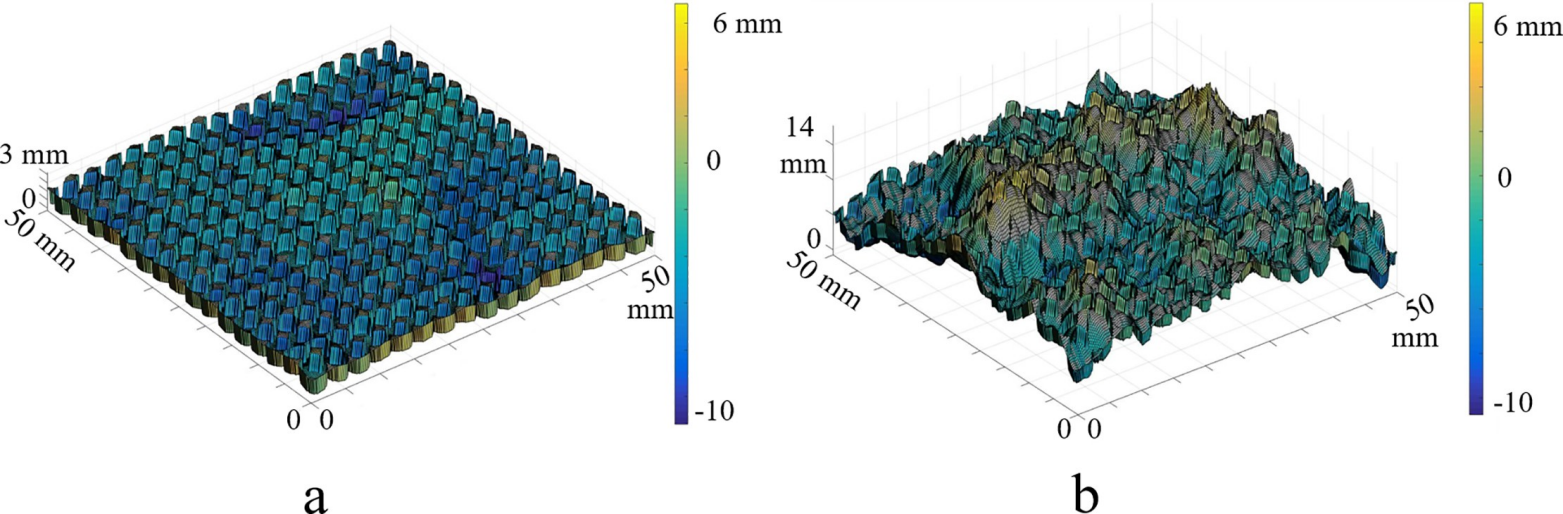
Profilometry images of fabricated specimens for parametric Level 1 (a) and Level 2 (b).

Because the *S*_*a*_, *S*_*v*_, and *S*_*mr*_ parameters are mathematically dependent and co-vary with each other, it was not possible to isolate the effects of each of them on algal attachment. Therefore, experimentation focused on the effect of surfaces described by the combination of these three parameters at two different levels. Two composite treatments were designed by grouping high and low values of each of these three surface parameters (Fig 3). The values of the treatment levels were defined with considering depth of flow in the floway environment, such that the designed tiles would have a height (3.8 cm) relative to the depth of the flow. The summary of the values of the designed treatment levels is presented in Table 2.

In order to observe the algal colonization process on the growth tiles, twelve replicates were modeled and fabricated for each treatment level combination, for a total of 24 tiles per trial and 72 tiles overall. Because the parameter values were derived from a distribution function, the replicates for each level were not exactly the same but had self-similar surface characteristics values for the surface parameters of *S*_*a*_, *S*_*v*_, and *S*_*mr*_.

All fabricated surfaces were inspected with a structured profilometer (VR-3200, Keyence, Osaka, Japan) with 20 nm resolution in the vertical direction to confirm the fidelity of the build. The results of profilometry of the inspected samples show that the *S*_*mr*_ had low average error percentage (5.4%), whereas the *S*_*a*_ and *S*_*v*_ had larger errors percentage, at 31.6% and 32.3%, respectively. The *S*_*mr*_ is more robust compared to *S*_*a*_ and *S*_*v*_ because it is more dependent on lower frequency variation (e.g., waviness) than the higher frequency roughness of the surface. In practice, it is generally easier to reproduce the waviness profilethan features in the roughness scale. [23].

### Validation experiment (steps j and k: algal inoculation)

**Fig 4:**
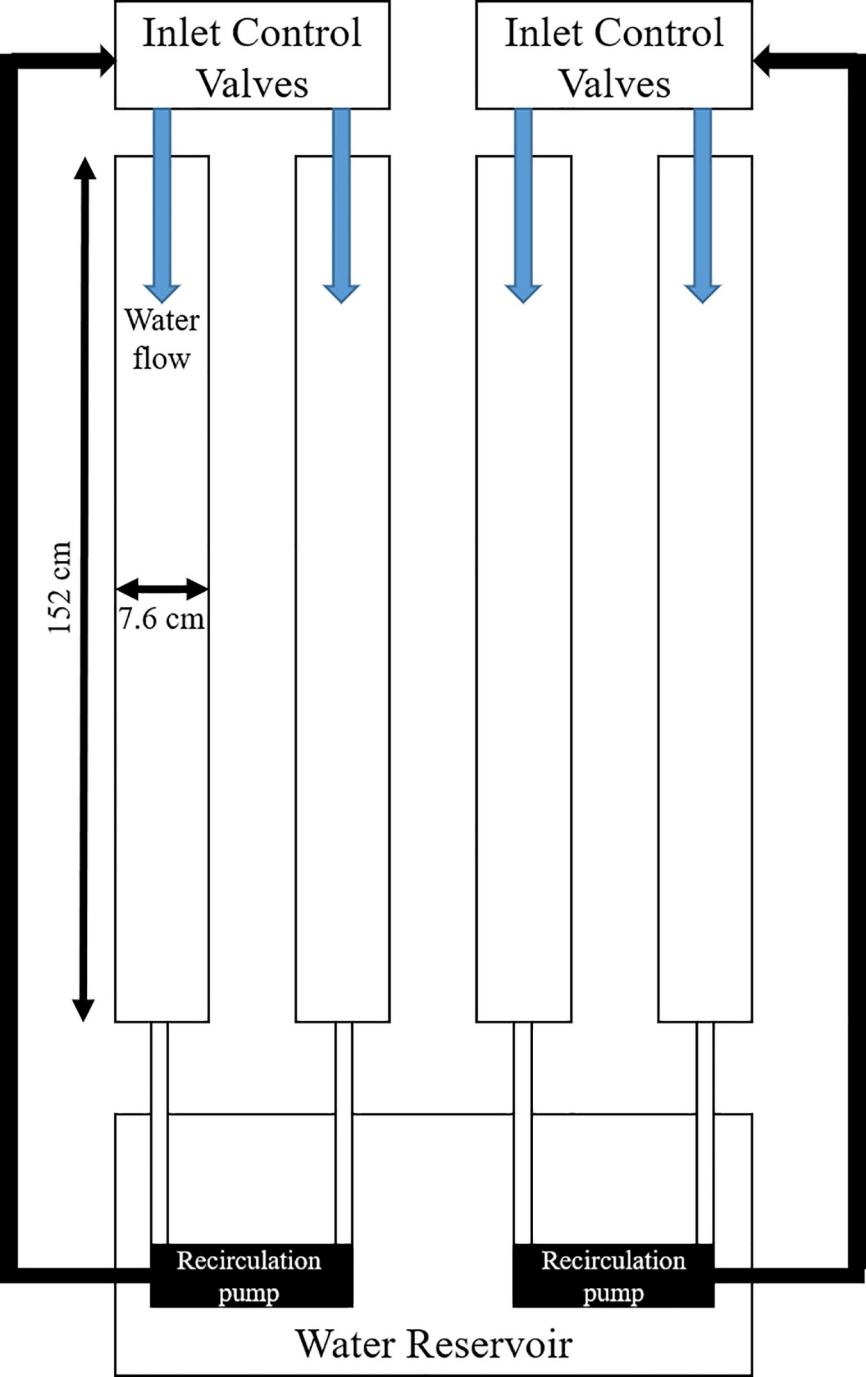
Plan view schematic representation of the four-lane floway photobioreactor system.

Growth tile replicates were exposed to a filamentous algae community in a 4-lane recirculating floway photobioreactor (Fig 4). The four lanes of the bioreactor system were constructed of PVC half round gutter material at a slope of 2.4%. Each lane of the bioreactor system had growth area dimensions of 152.4 cm x 7.6 cm. The growth area of each half round gutter was covered with a polyethylene screen mat with a 3 mm mesh gap (XV1672, Industrial Netting, Minneapolis, MN). All four lanes were supplied from and discharged to the same nutrient media reservoir.

Adjustable ball valves were used to regulate the flow rate at the inlet of each of the lanes. A collimator device comprising an array of 32 plastic tubes with a diameter of 12.7 mm each was installed at each channel inlet to straighten the flow.

The entire reactor system was inoculated with an algal community colonized on rock specimens that were collected from Chewacla Creek, Auburn, Alabama (32°32'51.0"N 85°28'53.7"W). Although twelve conspicuous green and diatom taxa were identified in the original *in situ* community using the illustrated key for identifying the genera of the common freshwater algae [33] and reported previously [9, 24], the floway photobioreactor was overwhelmingly dominated by *Mougeotia* species after 30 days of cultivation. A species of *Ulothrix* was also noted as present, as identified via microscopic analysis of multiple repeated samples from the floway system.

A total of three trial runs were conducted. In each trial, bare growth tiles were exposed to the algal community in the bioreactor for a period of seven days (168 hours). For each trial, twelve replicates were considered for each treatment level for a total of twenty-four, yielding in total 72 tile replicates across 3 trials. Prior to placement of growth tiles, algal biomass in the turf canopy was sacrificially harvested from the entire growing area of each of the flow lanes. Once the flow lanes were harvested, the tiles were placed in flow lanes. Each flow lane contained six sample tiles (three of each experimental level) alternately placed longitudinally, with equal spacing in between. The orientation of each tile placement relative to the direction of flow was randomly chosen.

Light for growth was provided by six 54W fluorescent light fixtures (Sun System Sun Blaze T5 High Output) with 8 bulbs each (Spectralux 901618, China) located directly above the 4-lane bioreactor system. The bioreactor system was operated under a continuous 24-hr light regime. The height and location of light fixtures were set at 1m from the water surface, providing moderate light intensity levels at the cultivation surface. Photosynthetic photon flux density, measured with a quantum flux probe (LI-190, LI-COR Biosciences, Lincoln, Nebraska, USA), on the algal growth substratum, averaged 219 ± 28 (range 157–268) μmol m^-2^ s^-1^ over the four flow lanes of floway bioreactor system.

The system was operated in a continuous mode by recirculating 57 L of freshwater nutrient solution to a common reservoir. Water flow was supplied to each lane by a separate centrifugal submersible pump (Supreme Mag Drive, Model MD 5, Danner Manufacturing, Islandia, New York, USA) submerged in the reservoir. Across the flow lanes, the mean flow rate was146 ± 2 cm^3^/s, and the mean water velocity, calculated as travel time down each lane was 29.3 (range 26.5–33.5) cm/s. The mean depth of the water flow in each lane was 3.8 cm. At these velocities, the estimated Reynolds number for open-channel flow averaged 6800, suggesting transitional flow characteristics.

During trial runs, nutrient media was renewed daily through replacement of half of the reservoir volume with fresh media. Growth media was a modified F/2 recipe (Proline, Pentair, Apopka, Fl), using nutrient and vitamin components mixed in fresh distilled water and diluted to approximately match nutrient concentration levels in stream waters. Water temperature, pH, and conductivity were monitored through daily sampling using a handheld combination pH/EC probe (HI 98130, Hanna Instruments, Woonsocket, Rhode Island) and portable reflectometer (RQflex plus 10, Sigma-Aldrich Corporation, St. Louis, Missouri). Throughout the experiment, dissolved N concentrations were measured as 1.74 ± 0.46 (range 0.90–2.71, n = 43) ppm NO_3_-N, water temperature as 75.1 ± 1.2 (range 70.7–76.8, n = 43) F, and pH as 6.87 ± 0.4 (range 5.42–7.63, n = 43).

Biomass accrual rate on the tiles was monitored throughout each trial by daily photographic imaging. Each tile was photographed in situ every 12 hours with a Canon EOS Rebel T5i DSLR camera (5184 x 3456 pixels) with 18–55 mm lens. The distance between the camera lens and samples was kept constant at 20 cm distance using a camera jig mount. The light intensity and illumination angle during the shooting process were kept constant and similar to the experimental conditions.

Total biomass on each tile was measured at the end of each trial period (t = 168 hr) by sacrificial harvesting and filtration. During this harvesting, the bioreactor system flow was stopped and drained. The growth tiles were gently removed, and any algae filaments attached to the underside were removed with a scraper and disposed of. The tiles were then submerged in 200 mL of distilled water inside resealable plastic zipper storage bags. Bagged samples were then placed in a sonicator bath (Branson 5800, Branson Ultrasonics Corporation, Danbury, Connecticut) and sonicated for 60 min at 40 kHz frequency to promote detachment. Remaining biomass still attached to the tiles after sonication was removed manually via brushing with a soft brush and rinsing with an additional 50 mL of distilled water. All biomass in the wash water was captured via vacuum filtration using 90 mm glass fiber filters (model Q8, Fisher Scientific, Pittsburgh, PA), and dry biomass was measured by gravimetric dry weight analysis at 105 C [34].

Biomass amounts on Level 1 and Level 2 substrata were compared statistically. An analysis of the underlying ANOVA assumptions for homoscedasticity was performed to ensure the validity of the approach. This included the Anderson-Darling test of normality and residual plot analysis, which showed normality on the data.

## Results and discussion

**Fig 5:**
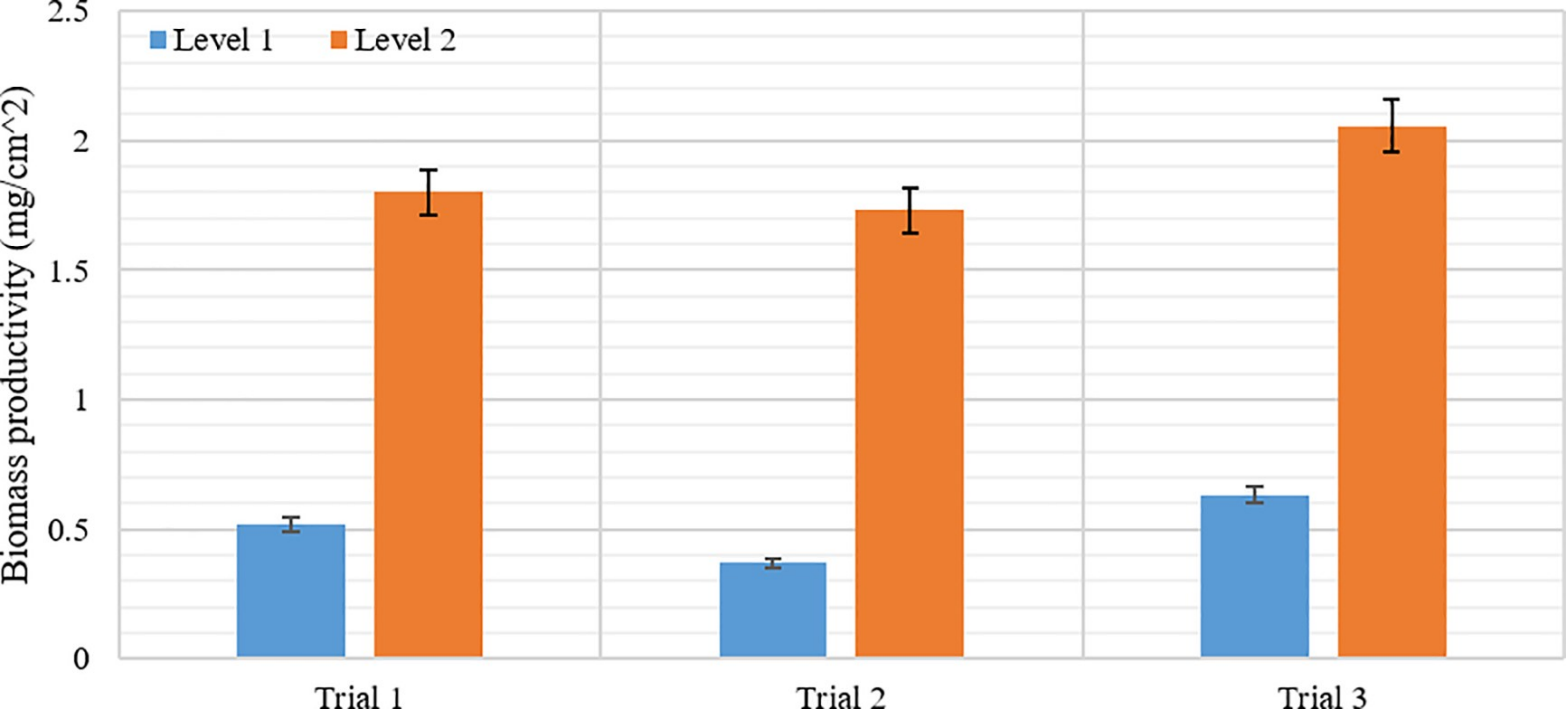
Algal biomass dry-weight at 168 hours (7 days) of growth.

Overall, across all trials and lanes, the total algal biomass accrued was substantially greater on Level 2 than on Level 1 substratum designs (Fig 5). The total biomass after seven days (168 hr) for Level 2 (1.86 ± 0.40 mg/cm^2^) was significantly (3.7 times) higher than that of Level 1 (0.50 ± 0.16 mg/cm^2^). The result of ANOVA testing showed that the main effect of the treatment was significant (F [1, 70] = 352.47, p < 0.001). Additionally, a Tukey test showed that there was no significant difference between flow lanes for each level.

Based on these results, the surface topography, as described by the parameters *S*_*a*_, *S*_*v*_, and *S*_*mr*_, significantly affects the colonization rate of the dominant algal species. The parametric analyses of natural substrata show that the *S*_*a*_, *S*_*v*_, and *S*_*mr*_ as combined promote surface attraction of freshwater algal species. The results of culture experiments show that the algae colonization rate responded to surfaces that were described by higher values of *S*_*a*_ (arithmetical mean height) and *S*_*v*_ (maximum pit height), and lower values of *S*_*mr*_ (peak material portion) parameters. In particular, results from the experiments show values that demonstrate greater algal colonization rates correlating with greater surface roughness and roughness element peakedness.

**Fig 6:**
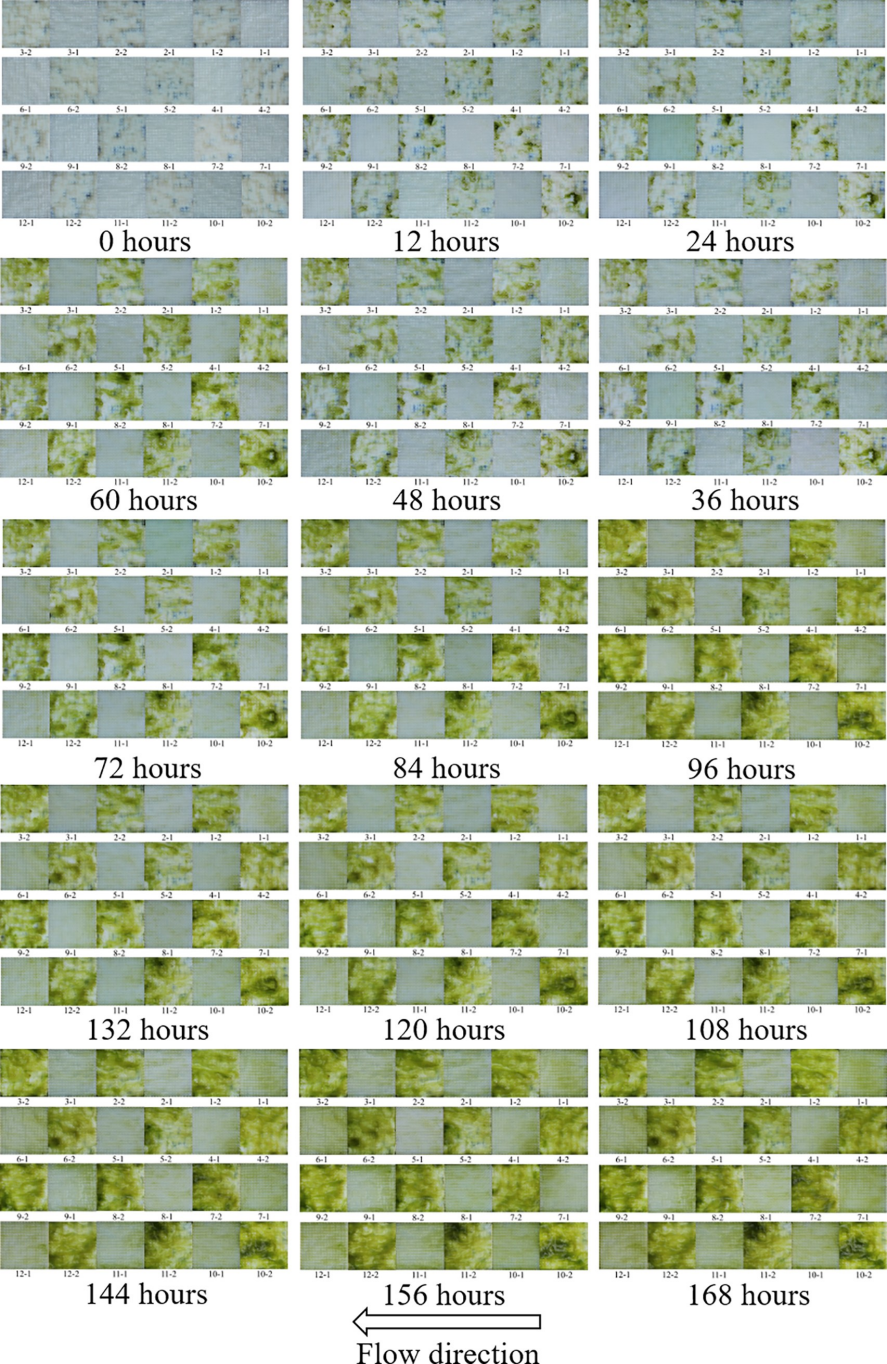
Attachment/settlement photographs at 12-hours intervals (trial 1, 168 hours) for all replicates at both levels.

In addition, the observations of colonization over the seven-day inoculation period appear to agree with an understanding of microbial colonization dynamics on surfaces, where roughness elements on substratum contribute to rates of early colonization through cell capture and hydrodynamic features at or near the scale of the cell [9]. Fig 6 shows the attached algae on the tiles (placed in an alternating layout of the two levels) from trial 1 for every 12 hours and throughout the 168 hours of the trial. Cursory inspection of Fig 6 indicates that there is a strong contrast in biomass coverage between Levels 1 and 2 as evidenced by the emerging alternating visual pattern throughout 7-day inoculation. Biomass appears to accrue rapidly on all Level 2 substratum and accrues comparatively slowly on Level 1 substratum.

The results of measured biomass are matched with the photographic observations throughout the seven-day inoculation progress, documenting the process of biomass accrual over the trial (Fig 6). Biomass is seen to emerge in specific areas on Level 2 tiles within the first 12 hours of exposure to the colonizing community, whereas biomass is conspicuously absent from Level 1 tiles for considerably longer in time. The localization of initial colonization suggests a correlation between topographic features and attraction of colonizing algal cells.

The process of colonization, as represented by the time sequence depicted in Fig 6, displays patterns of colonization, accrual, and expansion of biomass coverage over time that suggests the nature of the relationship between the surface features and the colonizing cells, specifically demonstrating the role of the topographical peaks on initiation of colonization. Comparing Level 2 to Level 1, a surface with a higher average roughness (that is, higher deviation from the mean surface plane) has, on average, taller hills and deeper dales that may help to increase the chance of entangling and thus trapping algal cells in flowing water. On average, the hills of Level 2 (average hills height = 8.29 mm, average dales depth = 8.35 mm) were 5.71 mm taller, and the dales were 1.71 mm deeper than those of Level 1 (average hills height = 2.58 mm, average dales depth = 6.64 mm).

**Fig 7:**
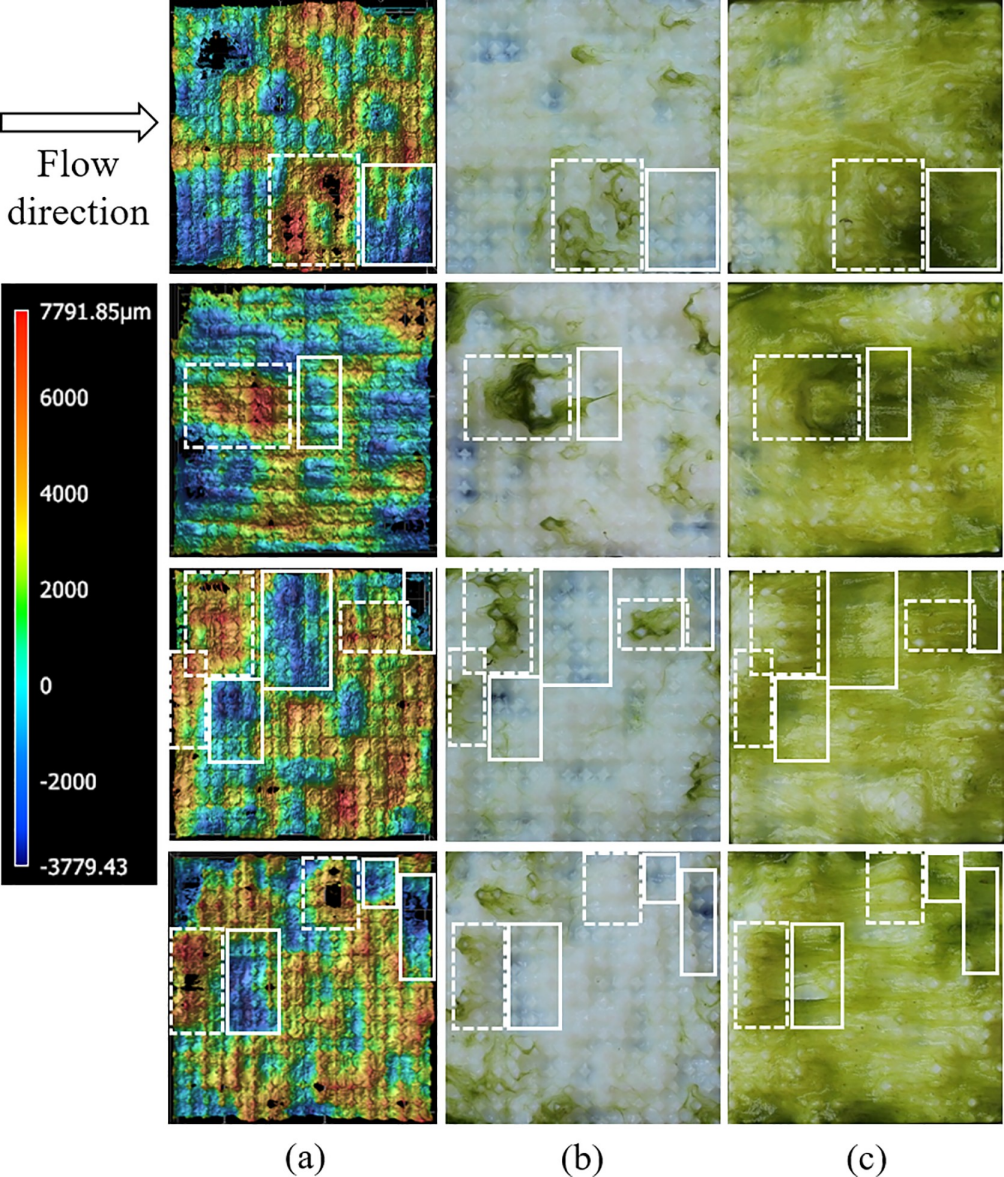
Profilometry heat maps (a) and photographs of colonization sequence at time 12 hours (b) and 120 hours (c) for four different samples (Level 2, trial 1).

The correlation of early algal colonization areas with those of high elevation hills is demonstrated upon further analysis of the colonization time sequence for Level 2 topographies. Fig 7 shows the photographic images of algae on treatment Level 2 after 12 hours of inoculation, and the corresponding profilometry heat maps for the same surfaces, taken with a wide-area 3D measurement system (VR-3200, Keyence, Osaka, Japan). Based on these observations, it can be hypothesized that the higher the maximum height of the surface profiles facing the flow direction, the higher the chances of trapping or coming into contact with algal cells and filaments. It can be observed that, after 12 hours of colonization, the primary locations for initial algal colonization were topographical features (hills) high above the mean plane. Furthermore, this effect appears to be more pronounced when the hills are followed by adjacent dales in the downstream direction (Fig 7). In order to quantify the effect of hills on early settlement, the ratio of the number of hills that appear to attract algae to those hills that do not was calculated. The features with the height of 3000 μm or higher in the profilometry images were considered as hills and the features with the height of zero or below were considered as dales. Based on these calculations, more than 98% of areas where algae were initially localized after 12 hours of inoculation were hills.

The time sequence of algal colonization can be related to the heterogeneity of the topography as described by the spatial distribution of hills and dales. Analysis of the hill/dale distribution and algal biomass presence for Level 2 topographies over the time is possible by analyzing the sequence of profilometry images and matching sample images throughout the inoculation process (Fig 7). As previously described, the hill sections show the greatest presence of algal biomass early in the colonization process, suggesting that algal cells or filaments are entrapped initially in these areas. This is consistent with known reproduction strategies of the dominant alga genus *Mougeotia*, which often forms new filamentous growth adhered to surfaces from small filament fragments broken off and carried downstream in the flow [35–36]. Once the filaments were entrapped, it can be expected that they contribute to additional biomass accrual on the surface through two concurrent processes—cellular growth and reproduction and capture or adsorption of other algal cells in the flow [34, 37–38]. As biomass accrues on the surface, the additional biomass over time extends into the downstream dale sections (solid rectangular sections in Fig 7), suggesting that the dale areas become refugia for biomass accrual processes. This trend is especially evident for hill sections and the adjacent downstream dales, where increased downstream biomass evident in later images of the time sequence is due to the quiescent conditions of the locale induced by lower drag and lift [39–40]. For example, as displayed in Fig 7 (colonization time: 12 hours), when the colonization process starts, algal biomass is present mostly at the hill section areas. Over time, algal biomass propagates into adjacent dale sections immediately downstream. Biomass continues to increase in the adjacent downstream dales (Fig 7, colonization time 120 hours), suggesting adsorption of more algal cells and continued growth and reproduction of already attached cells. This pattern was observed in all tile replicate images for Level 2 topography. This suggests that the distribution of hills and dales is key to rates of initial colonization and time-dependent accumulation of biomass, and optimum distributions of hill-dale height and spatial pattern for maximum rates of algal colonization and accrual likely exist, a potential focus for future research efforts.

## Conclusions

This work revealed a set of surface topography parameters (*S*_*a*_, *S*_*v*_, and *S*_*mr*_) that significantly correlated with presence of attached periphytic algae on natural rock substrata from a flow environment. Demonstration of the significance of these parameters was accomplished by filamentous algal colonization experiments on manufactured artificial substrata with two different parametric value ranges. The results showed significant increases in early biomass accrual rate on artificial substrata with higher values of topographic parameters. In addition, biomass accrual patterns followed the patterning of hill/dale distribution as aligned with predominant flow velocity vectors, where hills served as initial colonization areas and dales served as quiescent low-flow areas for expansion of the biomass. These results suggest that, for a given height distance profile on the substratum, flow condition, and colonizing periphyton community, an optimum areal distribution of hill and dale sequences exists that maximizes the rate of colonization and early sequence growth rates.

## Supporting information

S1 Data Log data for surface texture and significant features’ (dales) parameters of colonized and non-colonized areas. Log data from biomass measurement Log data from bioreactor Log data from computer model.
(DOCX)

S1 Appendix Nomenclature. (DOCX)
