# Supplementary material for: Engineering of bio-mimetic substratum topographies for enhanced early colonization of filamentous algae
Source: PLoS One. 2019 Jul 5;14(7):e0219150. doi: 10.1371/journal.pone.0219150 (PMC6611581; doi:10.1371/journal.pone.0219150)
Supplement: S1 Data — Log data from biomass measurement Log data from bioreactor Log data from computer model. (DOCX) [file pone.0219150.s001.docx]

Log data for surface texture and significant features’ (dales) parameters of colonized and non-colonized areas

| Type of parameter | Parameter | Description | Mean colonized | $\boldsymbol{\sigma}$ colonized | Mean non-colonized | $\boldsymbol{\sigma}$  non-colonized | p-value |
| --- | --- | --- | --- | --- | --- | --- | --- |
| Feature Parameters | *S_10z_ (µm)* | ten-point height of the surface | 430.9 | 95.9 | 441.9 | 174.8 | 0.633 |
|  | *S_5v_ (µm)* | five-point pit height of the surface | 217.7 | 50.2 | 234.9 | 127.3 | 0.832 |
| Height Parameters | *S_q_ (µm)* | root mean square height | 59.2 | 15.8 | 50.3 | 17.0 | 0.085 |
|  | *S_sk_* | skewness | -0.2 | 0.5 | -0.3 | 0.4 | 0.663 |
|  | *S_ku_* | kurtosis | 2.7 | 0.3 | 2.8 | 0.5 | 0.581 |
|  | *S_p_ (µm)* | maximum peak height | 207.2 | 91.3 | 192.6 | 75.6 | 0.542 |
|  | *S_v_ (µm)* | maximum pit height | 203.9 | 49.1 | 163.6 | 55.8 | 0.015 |
|  | *S_z_ (µm)* | maximum height of the surface | 424.9 | 114.8 | 367.4 | 147.3 | 0.136 |
|  | *S_a_ (µm)* | arithmetical mean of the absolute of the ordinate values | 46.6 | 12.0 | 38.2 | 10.9 | 0.026 |
| Functional Parameters | *S_mr_ (%)* | areal material ratio | 0.0008 | 0.0007 | 0.0040 | 0.0020 | 0.000 |
|  | *S_mc_ (µm)* | inverse areal material ratio | 87.8 | 41.3 | 104.7 | 55.2 | 0.224 |
|  | *S_xp_ (µm)* | peak extreme height | 133.0 | 49.1 | 115.4 | 46.5 | 0.222 |
| Spatial Parameters | *S_al_ (mm)* | autocorrelation length | 0.8 | 0.4 | 8.7 | 41.6 | 0.410 |
|  | *S_tr_* | texture aspect ratio | 0.5 | 0.2 | 0.4 | 0.2 | 0.150 |
|  | *S_td_ °* | texture direction of the scale-limited surface | 87.3 | 63.1 | 97.9 | 66.9 | 0.112 |
| Functional Parameters (Volume) | *V_m_ (mm³/mm²)* | material volume | 0.01 | 0.01 | 0.1 | 0.2 | 0.383 |
|  | *V_v_ (mm³/mm²)* | void volume | 0.09 | 0.03 | 2.2 | 10.9 | 0.392 |
|  | *V_mp_ (mm³/mm²)* | peak material volume | 0.01 | 0.01 | 0.04 | 0.2 | 0.382 |
|  | *V_mc_ (mm³/mm²)* | core material volume | 0.06 | 0.02 | 1.7 | 8.3 | 0.361 |
|  | *V_vc_ (mm³/mm²)* | core void volume | 0.08 | 0.03 | 2.1 | 10.3 | 0.424 |
|  | *V_vv_ (mm³/mm²)* | dale void volume | 0.01 | 0.00 | 0.12 | 0.6 | 0.383 |
| Functional Parameters (Stratified surfaces) | *S_k_ (µm)* | core height | 40.9 | 16.6 | 44.3 | 23.8 | 0.582 |
|  | *S_pk_ (µm)* | reduced peak height | 15.2 | 5.8 | 14.9 | 9.9 | 0.931 |
|  | *S_vk_ (µm)* | reduced dale height | 29.5 | 13.3 | 29.4 | 16.1 | 0.972 |
|  | *S_mr1_ (%)* | (peaks) ratio of the area of the material at the intersection line | 8.8 | 2.1 | 8.2 | 1.9 | 0.102 |
|  | *S_mr2_ (%)* | (dales) ratio of the area of the material at the intersection line | 86.5 | 3.7 | 86.1 | 3.2 | 0.68 |

**Log data from biomass measurement**

|  | Level 1 | | |
| --- | --- | --- | --- |
|  | Sample | Dried biomass-level 1 (gr) | (mg/cm^2^) |
| Trial 1 | 1_1 | 0.0149 | 0.596 |
|  | 2_1 | 0.0098 | 0.392 |
|  | 3_1 | 0.0133 | 0.532 |
|  | 4_1 | 0.0142 | 0.568 |
|  | 5_1 | 0.0112 | 0.448 |
|  | 6_1 | 0.016 | 0.64 |
|  | 7_1 | 0.0123 | 0.492 |
|  | 8_1 | 0.0083 | 0.332 |
|  | 9_1 | 0.0184 | 0.736 |
|  | 10_1 | 0.0122 | 0.488 |
|  | 11_1 | 0.0096 | 0.384 |
|  | 12_1 | 0.0154 | 0.616 |
| Trial 2 | 1_1 | 0.0065 | 0.26 |
|  | 2_1 | 0.0101 | 0.404 |
|  | 3_1 | 0.0128 | 0.512 |
|  | 4_1 | 0.0092 | 0.368 |
|  | 5_1 | 0.0072 | 0.288 |
|  | 6_1 | 0.013 | 0.52 |
|  | 7_1 | 0.0069 | 0.276 |
|  | 8_1 | 0.0051 | 0.204 |
|  | 9_1 | 0.0062 | 0.248 |
|  | 10_1 | 0.0127 | 0.508 |
|  | 11_1 | 0.0158 | 0.632 |
|  | 12_1 | 0.0056 | 0.224 |
| Trial 3 | 1_1 | 0.0123 | 0.492 |
|  | 2_1 | 0.0141 | 0.564 |
|  | 3_1 | 0.0138 | 0.552 |
|  | 4_1 | 0.0116 | 0.464 |
|  | 5_1 | 0.0126 | 0.504 |
|  | 6_1 | 0.0159 | 0.636 |
|  | 7_1 | 0.0211 | 0.844 |
|  | 8_1 | 0.0144 | 0.576 |
|  | 9_1 | 0.0174 | 0.696 |
|  | 10_1 | 0.0202 | 0.808 |
|  | 11_1 | 0.0171 | 0.684 |
|  | 12_1 | 0.0187 | 0.748 |
| Average | | 0.0127 | 0.5065556 |
| STD | | 0.0041 | 0.1658258 |
| Max | | 0.0211 | 0.844 |
| Min | | 0.0051 | 0.204 |
| Median | | 0.01275 | 0.51 |

|  | Level 2 | | |
| --- | --- | --- | --- |
|  | Sample | Dried biomass-level 2 | (mg/cm^2^) |
| Trial 1 | 1_1 | 0.0361 | 1.444 |
|  | 2_1 | 0.0339 | 1.356 |
|  | 3_1 | 0.0434 | 1.736 |
|  | 4_1 | 0.0415 | 1.66 |
|  | 5_1 | 0.0358 | 1.432 |
|  | 6_1 | 0.0354 | 1.416 |
|  | 7_1 | 0.0552 | 2.208 |
|  | 8_1 | 0.0495 | 1.98 |
|  | 9_1 | 0.0465 | 1.86 |
|  | 10_1 | 0.0663 | 2.652 |
|  | 11_1 | 0.0431 | 1.724 |
|  | 12_1 | 0.0532 | 2.128 |
| Trial 2 | 1_1 | 0.0401 | 1.604 |
|  | 2_1 | 0.0314 | 1.256 |
|  | 3_1 | 0.0541 | 2.164 |
|  | 4_1 | 0.0465 | 1.86 |
|  | 5_1 | 0.0292 | 1.168 |
|  | 6_1 | 0.0393 | 1.572 |
|  | 7_1 | 0.0646 | 2.584 |
|  | 8_1 | 0.04 | 1.6 |
|  | 9_1 | 0.0389 | 1.556 |
|  | 10_1 | 0.056 | 2.24 |
|  | 11_1 | 0.038 | 1.52 |
|  | 12_1 | 0.0416 | 1.664 |
| Trial 3 | 1_1 | 0.0474 | 1.896 |
|  | 2_1 | 0.0454 | 1.816 |
|  | 3_1 | 0.0514 | 2.056 |
|  | 4_1 | 0.0543 | 2.172 |
|  | 5_1 | 0.0543 | 2.172 |
|  | 6_1 | 0.0504 | 2.016 |
|  | 7_1 | 0.0398 | 1.592 |
|  | 8_1 | 0.0584 | 2.336 |
|  | 9_1 | 0.0572 | 2.288 |
|  | 10_1 | 0.0698 | 2.792 |
|  | 11_1 | 0.0523 | 2.092 |
|  | 12_1 | 0.0363 | 1.452 |
| Average | | 0.0466 | 1.862889 |
| STD | | 0.0100 | 0.400496 |
| Max | | 0.0698 | 2.792 |
| Min | | 0.0292 | 1.168 |
| Median | | 0.04595 | 1.838 |

## **Log data from bioreactor**

| Date | Time | Room temperature (F) | Water Temperature (F) | pH | Conductivity | Nitrate (mg/l NO3^-) | Nitrate (mg/l NO3-N) | Comments |
| --- | --- | --- | --- | --- | --- | --- | --- | --- |
| 12/3/2017 | 6:17 PM | 70.70 | 73.00 | 5.42 | 0.03 | 10.00 | 2.26 | Trial 1 |
| 12/4/2017 | 8:32 AM | 70.70 | 76.40 | 7.57 | 0.03 | 12.00 | 2.71 |  |
|  | 8:17 PM | 69.70 | 76.40 | 7.42 | 0.03 | 12.00 | 2.71 |  |
| 12/5/2017 | 8:37 AM | 70.00 | 74.60 | 7.63 | 0.02 | 8.00 | 1.81 |  |
|  | 8:17 PM | 70.20 | 76.60 | 7.55 | 0.03 | 9.00 | 2.03 |  |
| 12/6/2017 | 8:31 AM | 68.90 | 75.70 | 7.26 | 0.03 | 8.00 | 1.81 |  |
|  | 8:25 PM | 67.00 | 75.30 | 7.57 | 0.03 | 8.00 | 1.81 |  |
| 12/7/2017 | 8:33 AM | 64.80 | 75.20 | 6.89 | 0.03 | 6.00 | 1.36 |  |
|  | 8:03 PM | 69.20 | 75.30 | 6.60 | 0.03 | 6.00 | 1.36 |  |
| 12/8/2017 | 8:44 AM | 67.50 | 75.20 | 7.24 | 0.03 | 6.00 | 1.36 |  |
|  | 8:59 PM | 68.00 | 75.20 | 6.85 | 0.03 | 7.00 | 1.58 |  |
| 12/9/2017 | 8:43 AM | 67.70 | 75.00 | 6.40 | 0.03 | 7.00 | 1.58 |  |
|  | 8:29 PM | 61.80 | 74.40 | 6.86 | 0.03 | 5.00 | 1.13 |  |
| 12/10/2017 | 8:39 AM | 64.80 | 74.60 | 7.24 | 0.03 | 6.00 | 1.36 |  |
|  | 7:00 PM | 66.10 | 74.00 | 7.16 | 0.03 | 4.00 | 0.90 | Trial 2 |
| 12/11/2017 | 8:44 AM | 66.80 | 74.80 | 6.80 | 0.03 | 5.00 | 1.13 |  |
|  | 8:35 PM | 66.40 | 75.00 | 7.05 | 0.03 | 4.00 | 0.90 |  |
| 12/12/2017 | 8:29 AM | 65.70 | 75.20 | 6.42 | 0.03 | 5.00 | 1.13 |  |
|  | 9:00 PM | 66.00 | 75.00 | 6.73 | 0.03 | 8.00 | 1.81 |  |
| 12/13/2017 | 10:06 AM | 62.70 | 74.30 | 6.20 | 0.03 | 8.00 | 1.81 |  |
|  | 9:25 PM | 67.70 | 74.00 | 6.80 | 0.03 | 7.00 | 1.58 |  |
| 12/14/2017 | 8:37 AM | 63.00 | 74.60 | 6.72 | 0.03 | 8.00 | 1.81 |  |
|  | 8:31 PM | 63.00 | 74.70 | 6.82 | 0.03 | 8.00 | 1.81 |  |
| 12/15/2017 | 8:41 AM | 65.80 | 70.70 | 6.86 | 0.03 | 9.00 | 2.03 |  |
|  | 8:18 PM | 65.50 | 74.40 | 6.67 | 0.03 | 8.00 | 1.81 |  |
| 12/16/2017 | 7:30 AM | 63.80 | 74.60 | 6.69 | 0.03 | 10.00 | 2.26 |  |
|  | 8:25 PM | 66.50 | 74.60 | 6.84 | 0.03 | 7.00 | 1.58 |  |
| 12/17/2017 | 8:12 AM | 66.10 | 74.70 | 6.87 | 0.03 | 9.00 | 2.03 |  |
|  | 5:15 PM | 66.80 | 76.10 | 6.41 | 0.03 | 8.00 | 1.81 | Trial 3 |
| 12/18/2017 | 8:25 AM | 68.90 | 76.50 | 6.90 | 0.03 | 10.00 | 2.26 |  |
|  | 8:21 PM | 68.10 | 76.00 | 6.96 | 0.03 | 8.00 | 1.81 |  |
| 12/19/2017 | 8:21 AM | 67.30 | 76.80 | 6.86 | 0.03 | 10.00 | 2.26 |  |
|  | 8:34 PM | 68.00 | 76.70 | 6.87 | 0.03 | 9.00 | 2.03 |  |
| 12/20/2017 | 8:47 AM | 68.10 | 76.80 | 6.55 | 0.03 | 8.00 | 1.81 |  |
|  | 8:31 PM | 67.90 | 77.00 | 6.58 | 0.03 | 8.00 | 1.81 |  |
| 12/21/2017 | 8:31 AM | 67.90 | 76.80 | 6.94 | 0.03 | 8.00 | 1.81 |  |
|  | 8:19 PM | 68.20 | 76.60 | 6.81 | 0.03 | 7.00 | 1.58 |  |
| 12/22/2017 | 8:25 AM | 67.90 | 77.00 | 6.30 | 0.03 | 8.00 | 1.81 |  |
|  | 8:26 PM | 68.10 | 76.90 | 6.30 | 0.03 | 5.00 | 1.13 |  |
| 12/23/2017 | 8:23 AM | 68.40 | 77.30 | 6.94 | 0.03 | 8.00 | 1.81 |  |
|  | 7:42 PM | 68.50 | 77.00 | 6.38 | 0.03 | 7.00 | 1.58 |  |
| 12/24/2017 | 8:19 AM | 67.50 | 76.10 | 6.62 | 0.03 | 7.00 | 1.58 |  |
|  | 5:52 PM | 67.00 | 75.80 | 6.93 | 0.03 | 10.00 | 2.26 |  |


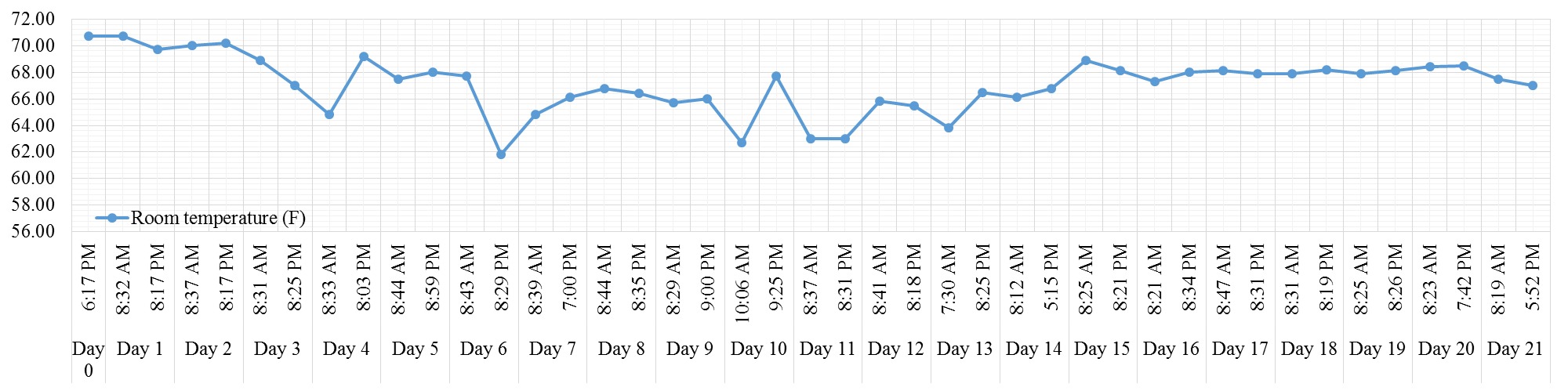


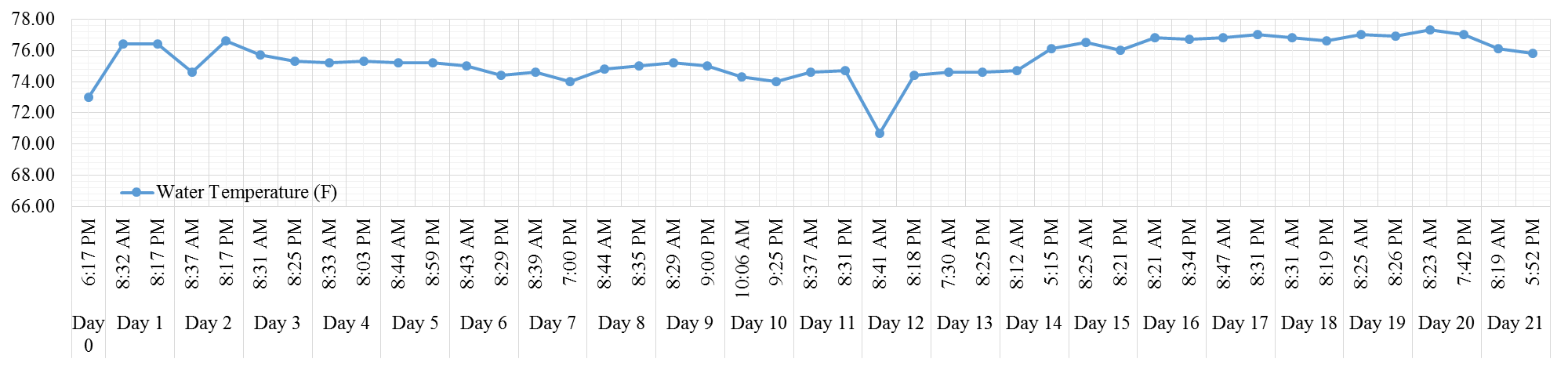


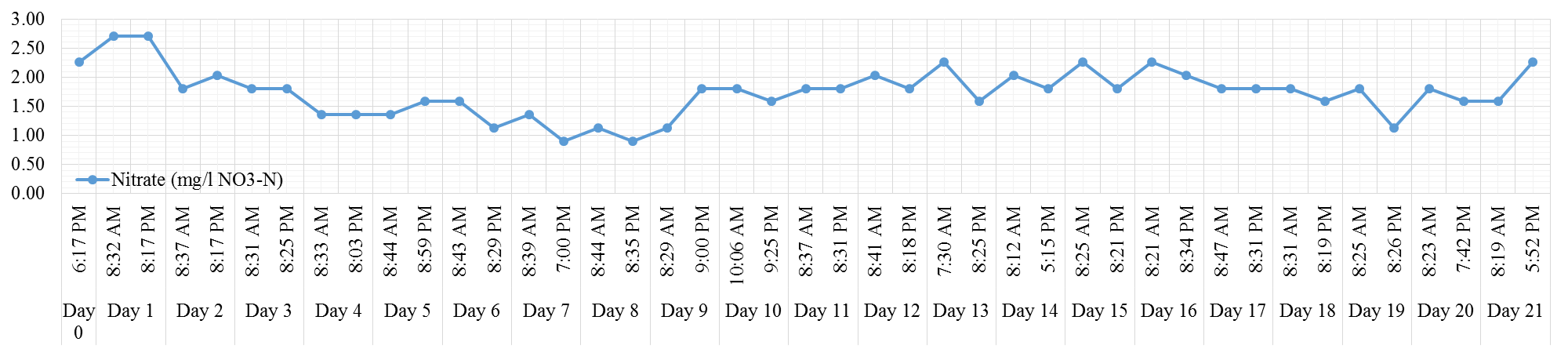


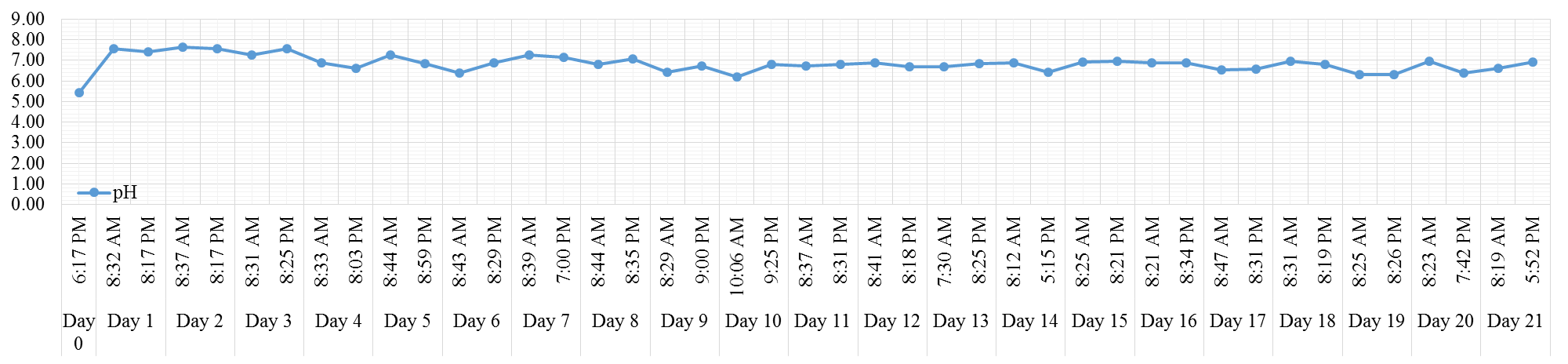


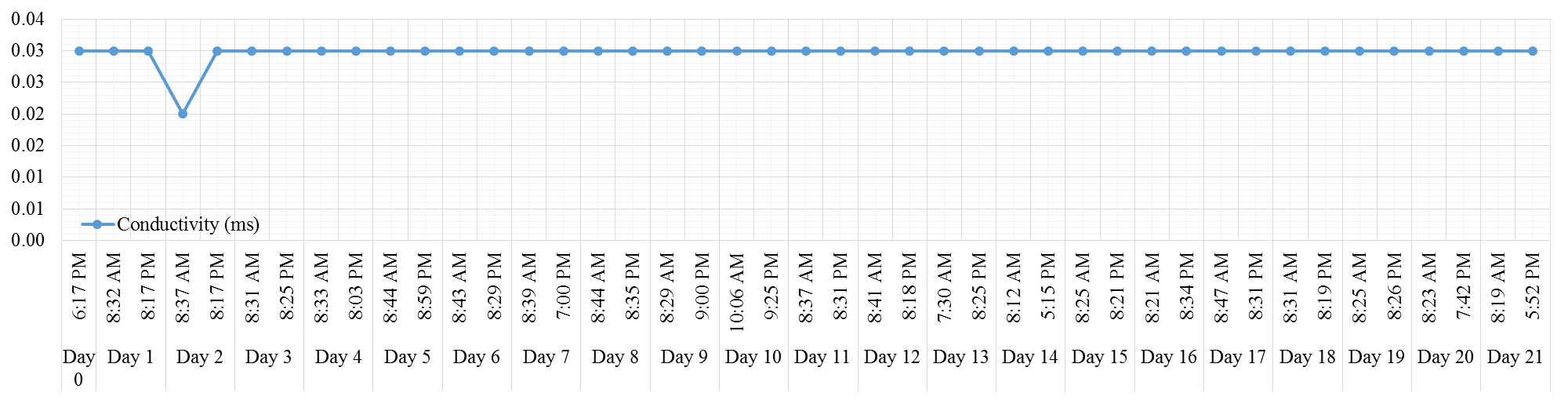


|  | Time to fill 2 litters (s) | | | |
| --- | --- | --- | --- | --- |
|  | Lane 1 | Lane 2 | Lane 3 | Lane 4 |
| Rep 1 | 13.980 | 13.560 | 13.510 | 13.500 |
| Rep 2 | 13.860 | 13.830 | 13.570 | 13.780 |
| Rep 3 | 14.130 | 13.680 | 13.730 | 13.400 |
| Rep 4 | 13.960 | 13.660 | 13.730 | 13.520 |
| Rep 5 | 13.880 | 13.530 | 13.910 | 13.450 |
|  |  |  |  |  |
| Average | 13.962 | 13.652 | 13.690 | 13.530 |
| STD | 0.107 | 0.118 | 0.157 | 0.147 |

|  | Flow rate (m^3/s) | | | |
| --- | --- | --- | --- | --- |
|  | Lane 1 | Lane 2 | Lane 3 | Lane 4 |
| Rep 1 | 0.0001431 | 0.0001475 | 0.0001480 | 0.0001481 |
| Rep 2 | 0.0001443 | 0.0001446 | 0.0001474 | 0.0001451 |
| Rep 3 | 0.0001415 | 0.0001462 | 0.0001457 | 0.0001493 |
| Rep 4 | 0.0001433 | 0.0001464 | 0.0001457 | 0.0001479 |
| Rep 5 | 0.0001441 | 0.0001478 | 0.0001438 | 0.0001487 |
|  |  |  |  |  |
| Average | 0.0001433 | 0.0001465 | 0.0001461 | 0.0001478 |
| STD | 0.0000011 | 0.0000013 | 0.0000017 | 0.0000016 |

|  | Travel time (s) | | | |
| --- | --- | --- | --- | --- |
|  | Lane 1 | Lane 2 | Lane 3 | Lane 4 |
| Rep 1 | 3.620 | 4.340 | 4.050 | 4.640 |
| Rep 2 | 3.610 | 4.250 | 4.280 | 4.470 |
| Rep 3 | 3.620 | 4.360 | 4.190 | 4.140 |
| Rep 4 | 3.630 | 4.130 | 4.140 | 4.920 |
| Rep 5 | 3.610 | 4.220 | 4.490 | 4.900 |
|  |  |  |  |  |
| Average | 3.618 | 4.260 | 4.230 | 4.614 |
| STD | 0.008 | 0.094 | 0.167 | 0.325 |

|  | Velocity (m/s) | | | |
| --- | --- | --- | --- | --- |
|  | Lane 1 | Lane 2 | Lane 3 | Lane 4 |
| Rep 1 | 0.334 | 0.281 | 0.299 | 0.263 |
| Rep 2 | 0.335 | 0.287 | 0.283 | 0.273 |
| Rep 3 | 0.334 | 0.280 | 0.289 | 0.295 |
| Rep 4 | 0.333 | 0.295 | 0.292 | 0.248 |
| Rep 5 | 0.335 | 0.289 | 0.269 | 0.249 |
| Length (m) | 1.210 | 1.220 | 1.210 | 1.220 |
|  |  |  |  |  |
| Average | 0.334 | 0.286 | 0.286 | 0.265 |
| STD | 0.001 | 0.006 | 0.011 | 0.019 |

## **Log data from computer model**

| Reps | Level 1 | | | | | | | | |
| --- | --- | --- | --- | --- | --- | --- | --- | --- | --- |
|  | Zavg | Sa | Sq | Ssk | Sku | Sv | Sp | Sz | Smr |
| Rep 1 | -1.62 | 0.74 | 0.92 | 0.98 | 2.81 | 6.68 | 2.83 | 9.51 | 31.67 |
| Rep 2 | -1.43 | 0.73 | 0.92 | 0.96 | 2.81 | 6.63 | 2.52 | 9.14 | 31.16 |
| Rep 3 | -1.65 | 0.73 | 0.91 | 0.98 | 2.76 | 6.69 | 2.54 | 9.24 | 30.07 |
| Rep 4 | -1.52 | 0.73 | 0.92 | 0.95 | 2.82 | 6.77 | 2.52 | 9.29 | 32.07 |
| Rep 5 | -1.66 | 0.74 | 0.93 | 0.93 | 2.85 | 7.16 | 2.75 | 9.91 | 31.06 |
| Rep 6 | -1.53 | 0.74 | 0.92 | 1.01 | 2.82 | 6.55 | 2.74 | 9.28 | 31.62 |
| Rep 7 | -1.47 | 0.72 | 0.91 | 0.98 | 2.79 | 6.71 | 2.51 | 9.22 | 29.05 |
| Rep 8 | -1.42 | 0.72 | 0.90 | 1.00 | 2.79 | 6.47 | 2.43 | 8.90 | 29.96 |
| Rep 9 | -1.56 | 0.71 | 0.89 | 1.05 | 2.77 | 6.35 | 2.56 | 8.91 | 28.88 |
| Rep 10 | -1.53 | 0.72 | 0.91 | 0.98 | 2.81 | 6.61 | 2.80 | 9.42 | 30.92 |
| Rep 11 | -1.62 | 0.73 | 0.92 | 0.95 | 2.82 | 6.65 | 2.68 | 9.33 | 31.80 |
| Rep 12 | -1.70 | 0.73 | 0.91 | 0.99 | 2.79 | 6.70 | 2.60 | 9.30 | 31.52 |
| Rep 13 | -1.42 | 0.72 | 0.90 | 1.00 | 2.79 | 6.47 | 2.43 | 8.90 | 29.96 |
| Rep 14 | -1.56 | 0.71 | 0.89 | 1.05 | 2.77 | 6.35 | 2.56 | 8.91 | 28.88 |
| Rep 15 | -1.51 | 0.71 | 0.90 | 1.04 | 2.81 | 6.53 | 2.43 | 8.96 | 28.21 |
| Rep 16 | -1.60 | 0.72 | 0.90 | 1.05 | 2.79 | 6.36 | 2.58 | 8.94 | 29.98 |
| Rep 17 | -1.65 | 0.73 | 0.91 | 0.98 | 2.76 | 6.69 | 2.54 | 9.24 | 30.07 |
| Rep 18 | -1.66 | 0.74 | 0.93 | 0.93 | 2.85 | 7.16 | 2.75 | 9.91 | 31.06 |
| Rep 19 | -1.47 | 0.72 | 0.91 | 0.98 | 2.79 | 6.71 | 2.51 | 9.22 | 29.05 |
| Rep 20 | -1.42 | 0.72 | 0.90 | 1.00 | 2.79 | 6.47 | 2.43 | 8.90 | 29.96 |
| Rep 21 | -1.56 | 0.71 | 0.89 | 1.05 | 2.77 | 6.35 | 2.56 | 8.91 | 28.88 |
| Rep 22 | -1.53 | 0.72 | 0.91 | 0.98 | 2.81 | 6.61 | 2.80 | 9.42 | 30.92 |
| Rep 23 | -1.53 | 0.73 | 0.92 | 0.97 | 2.79 | 6.81 | 2.59 | 9.40 | 31.02 |
| Rep 24 | -1.51 | 0.71 | 0.90 | 1.04 | 2.81 | 6.53 | 2.43 | 8.96 | 28.21 |
| Rep 25 | -1.65 | 0.73 | 0.91 | 0.98 | 2.76 | 6.69 | 2.54 | 9.24 | 30.07 |
| Rep 26 | -1.66 | 0.74 | 0.93 | 0.93 | 2.85 | 7.16 | 2.75 | 9.91 | 31.06 |
| Rep 27 | -1.47 | 0.72 | 0.91 | 0.98 | 2.79 | 6.71 | 2.51 | 9.22 | 29.05 |
| Rep 28 | -1.42 | 0.72 | 0.90 | 1.00 | 2.79 | 6.47 | 2.43 | 8.90 | 29.96 |
| Rep 29 | -1.56 | 0.71 | 0.89 | 1.05 | 2.77 | 6.35 | 2.56 | 8.91 | 28.88 |
| Rep 30 | -1.53 | 0.72 | 0.91 | 0.98 | 2.81 | 6.61 | 2.80 | 9.42 | 30.92 |
| Rep 31 | -1.51 | 0.71 | 0.90 | 1.04 | 2.81 | 6.53 | 2.43 | 8.96 | 28.21 |
| Rep 32 | -1.65 | 0.73 | 0.91 | 0.98 | 2.76 | 6.69 | 2.54 | 9.24 | 30.07 |
| Rep 33 | -1.66 | 0.74 | 0.93 | 0.93 | 2.85 | 7.16 | 2.75 | 9.91 | 31.06 |
| Rep 34 | -1.47 | 0.72 | 0.91 | 0.98 | 2.79 | 6.71 | 2.51 | 9.22 | 29.05 |
| Rep 35 | -1.51 | 0.71 | 0.90 | 1.04 | 2.81 | 6.53 | 2.43 | 8.96 | 28.21 |
| Rep 36 | -1.56 | 0.71 | 0.89 | 1.05 | 2.77 | 6.35 | 2.56 | 8.91 | 28.88 |
|  |  |  |  |  |  |  |  |  |  |
| Average | -1.55 | 0.72 | 0.91 | 0.99 | 2.80 | 6.64 | 2.58 | 9.22 | 30.04 |
| STD | 0.083563 | 0.01 | 0.011313 | 0.037362 | 0.025987 | 0.23 | 0.128491 | 0.31066 | 1.16 |
| CV (%) | 5.40 | 1.35 | 1.24 | 3.76 | 0.93 | 3.44 | 4.98 | 3.37 | 3.87 |
| Min | -1.70 | 0.71 | 0.89 | 0.93 | 2.76 | 6.35 | 2.43 | 8.90 | 28.21 |
| Max | -1.42 | 0.74 | 0.93 | 1.05 | 2.85 | 7.16 | 2.83 | 9.91 | 32.07 |

| Reps | Level 2 | | | | | | | | |
| --- | --- | --- | --- | --- | --- | --- | --- | --- | --- |
|  | Zavg | Sa | Sq | Ssk | Sku | Sv | Sp | Sz | Smr |
| Rep 1 | -1.92 | 2.17 | 2.74 | -0.09 | 3.00 | 8.84 | 9.71 | 18.56 | 51.86 |
| Rep 2 | -0.84 | 2.03 | 2.52 | -0.23 | 2.78 | 7.78 | 7.62 | 15.40 | 52.72 |
| Rep 3 | -1.10 | 2.18 | 2.73 | -0.25 | 3.20 | 10.34 | 8.44 | 18.78 | 50.90 |
| Rep 4 | -2.19 | 1.89 | 2.38 | -0.13 | 2.91 | 8.17 | 7.81 | 15.97 | 50.84 |
| Rep 5 | -0.72 | 2.07 | 2.54 | 0.00 | 2.60 | 7.97 | 8.03 | 16.01 | 50.65 |
| Rep 6 | -1.39 | 2.07 | 2.57 | -0.13 | 2.84 | 8.67 | 7.66 | 16.33 | 50.88 |
| Rep 7 | -1.67 | 2.41 | 2.96 | -0.20 | 2.68 | 9.58 | 8.45 | 18.03 | 52.25 |
| Rep 8 | -0.95 | 2.12 | 2.62 | -0.06 | 2.57 | 6.90 | 8.21 | 15.10 | 51.68 |
| Rep 9 | -1.08 | 1.87 | 2.35 | -0.26 | 2.93 | 8.31 | 7.56 | 15.87 | 53.75 |
| Rep 10 | -1.35 | 2.28 | 2.87 | -0.02 | 3.07 | 8.91 | 9.62 | 18.53 | 50.74 |
| Rep 11 | -1.89 | 2.24 | 2.78 | 0.06 | 2.76 | 8.33 | 9.00 | 17.34 | 50.54 |
| Rep 12 | -1.41 | 2.12 | 2.63 | -0.13 | 2.72 | 8.10 | 7.77 | 15.87 | 51.52 |
| Rep 13 | -1.92 | 2.17 | 2.74 | -0.09 | 3.00 | 8.84 | 9.71 | 18.56 | 51.86 |
| Rep 14 | -0.84 | 2.03 | 2.52 | -0.23 | 2.78 | 7.78 | 7.62 | 15.40 | 52.72 |
| Rep 15 | -1.10 | 2.18 | 2.73 | -0.25 | 3.20 | 10.34 | 8.44 | 18.78 | 50.90 |
| Rep 16 | -2.19 | 1.89 | 2.38 | -0.13 | 2.91 | 8.17 | 7.81 | 15.97 | 50.84 |
| Rep 17 | -0.72 | 2.07 | 2.54 | 0.00 | 2.60 | 7.97 | 8.03 | 16.01 | 50.65 |
| Rep 18 | -1.39 | 2.07 | 2.57 | -0.13 | 2.84 | 8.67 | 7.66 | 16.33 | 50.88 |
| Rep 19 | -1.67 | 2.41 | 2.96 | -0.20 | 2.68 | 9.58 | 8.45 | 18.03 | 52.25 |
| Rep 20 | -0.95 | 2.12 | 2.62 | -0.06 | 2.57 | 6.90 | 8.21 | 15.10 | 51.68 |
| Rep 21 | -1.08 | 1.87 | 2.35 | -0.26 | 2.93 | 8.31 | 7.56 | 15.87 | 53.75 |
| Rep 22 | -1.35 | 2.28 | 2.87 | -0.02 | 3.07 | 8.91 | 9.62 | 18.53 | 50.74 |
| Rep 23 | -1.89 | 2.24 | 2.78 | 0.06 | 2.76 | 8.33 | 9.00 | 17.34 | 50.54 |
| Rep 24 | -1.41 | 2.12 | 2.63 | -0.13 | 2.72 | 8.10 | 7.77 | 15.87 | 51.52 |
| Rep 25 | -1.92 | 2.17 | 2.74 | -0.09 | 3.00 | 8.84 | 9.71 | 18.56 | 51.86 |
| Rep 26 | -0.84 | 2.03 | 2.52 | -0.23 | 2.78 | 7.78 | 7.62 | 15.40 | 52.72 |
| Rep 27 | -1.67 | 2.41 | 2.96 | -0.20 | 2.68 | 9.58 | 8.45 | 18.03 | 52.25 |
| Rep 28 | -0.95 | 2.12 | 2.62 | -0.06 | 2.57 | 6.90 | 8.21 | 15.10 | 51.68 |
| Rep 29 | -1.08 | 1.87 | 2.35 | -0.26 | 2.93 | 8.31 | 7.56 | 15.87 | 53.75 |
| Rep 30 | -1.41 | 2.12 | 2.63 | -0.13 | 2.72 | 8.10 | 7.77 | 15.87 | 51.52 |
| Rep 31 | -0.77 | 1.88 | 2.32 | -0.16 | 2.69 | 6.81 | 7.10 | 13.90 | 51.44 |
| Rep 32 | -2.02 | 2.04 | 2.55 | -0.19 | 2.78 | 7.94 | 8.65 | 16.59 | 52.57 |
| Rep 33 | -1.61 | 2.34 | 2.89 | -0.31 | 2.71 | 8.36 | 8.18 | 16.54 | 53.05 |
| Rep 34 | -0.98 | 2.33 | 2.91 | -0.15 | 2.86 | 8.67 | 10.06 | 18.73 | 52.43 |
| Rep 35 | -1.65 | 1.98 | 2.52 | -0.15 | 3.16 | 8.24 | 8.28 | 16.52 | 51.21 |
| Rep 36 | -1.31 | 1.81 | 2.23 | -0.14 | 2.73 | 7.17 | 7.10 | 14.26 | 51.23 |
|  |  |  |  |  |  |  |  |  |  |
| Average | -1.37 | 2.11 | 2.63 | -0.14 | 2.82 | 8.35 | 8.29 | 16.64 | 51.73 |
| STD | 0.439486 | 0.16 | 0.199729 | 0.093453 | 0.17743 | 0.86 | 0.793722 | 1.402544 | 0.94 |
| CV (%) | 32.12 | 7.74 | 7.60 | 67.55 | 6.28 | 10.33 | 9.57 | 8.43 | 1.82 |
| Min | -2.19 | 1.81 | 2.23 | -0.31 | 2.57 | 6.81 | 7.10 | 13.90 | 50.54 |
| Max | -0.72 | 2.41 | 2.96 | 0.06 | 3.20 | 10.34 | 10.06 | 18.78 | 53.75 |
