# Supplementary material for: Engineering of bio-mimetic substratum topographies for enhanced early colonization of filamentous algae
Source: PLoS One. 2019 Jul 5;14(7):e0219150. doi: 10.1371/journal.pone.0219150 (PMC6611581; doi:10.1371/journal.pone.0219150)
Supplement: S1 Appendix — (DOCX) [file pone.0219150.s002.docx]

**S1 Appendix**

| Nomenclature |  |  |
| --- | --- | --- |
| Type of parameter | Parameter | Description |
|  | *S_10z_ (µm)* | ten-point height of the surface |
|  | *S_5v_ (µm)* | five-point pit height of the surface |
| Height Parameters | *S_q_ (µm)* | root mean square height |
|  | *S_sk_* | skewness |
|  | *S_ku_* | kurtosis |
|  | *S_p_ (µm)* | maximum peak height |
|  | *S_v_ (µm)* | maximum pit depth |
|  | *S_z_ (µm)* | maximum height of the surface |
|  | *S_a_ (µm)* | arithmetic mean of the absolute of the ordinate values within a defined area |
| Functional Parameters | *S_mr_ (%)* | areal material ratio |
|  | *S_mc_ (µm)* | inverse areal material ratio |
|  | *S_xp_ (µm)* | peak extreme height |
| Spatial Parameters | *S_al_ (mm)* | autocorrelation length |
|  | *S_tr_* | texture aspect ratio |
|  | *S_td_ °* | texture direction of the scale-limited surface |
| Functional Parameters (Volume) | *V_m_ (mm³/mm²)* | material volume |
|  | *V_v_ (mm³/mm²)* | void volume |
|  | *V_mp_ (mm³/mm²)* | peak material volume |
|  | *V_mc_ (mm³/mm²)* | core material volume |
|  | *V_vc_ (mm³/mm²)* | core void volume |
|  | *V_vv_ (mm³/mm²)* | dale void volume |
| Functional Parameters (Stratified surfaces) | *S_k_ (µm)* | core height |
|  | *S_pk_ (µm)* | reduced peak height |
|  | *S_vk_ (µm)* | reduced dale height |
|  | *S_mr1_ (%)* | (peaks) ratio of the area of the material at the intersection line |
|  | *S_mr2_ (%)* | (dales) ratio of the area of the material at the intersection line |
